# Persistent Proinflammatory Cytokine Profile in the Tear Fluid of Stable Keratoconus: Rethinking Clinical Quiescence

**DOI:** 10.1167/tvst.14.9.31

**Published:** 2025-09-22

**Authors:** Pedro Gil, João Quadrado Gil, Nuno Cruz, Celso Costa, Paulo Rodrigues-Santos, Luana Madalena Sousa, Jani Sofia Almeida, Rosa Fernandes, Nuno Alves, Andreia Rosa, Joaquim Murta

**Affiliations:** 1 University of Coimbra, Faculty of Medicine, Coimbra, Portugal; 2 São José Local Health Unit, Lisbon, Portugal; 3 Coimbra Local Health Unit, Coimbra, Portugal; 4 Clinical Academic Center of Coimbra (CACC), Coimbra, Portugal; 5 Immunology and Oncology Laboratory, Center for Neuroscience and Cell Biology (CNC), University of Coimbra, Coimbra, Portugal; 6 Institute of Immunology, Faculty of Medicine, University of Coimbra, Coimbra, Portugal; 7 Center for Investigation in Environment, Genetics and Oncobiology (CIMAGO), University of Coimbra, Coimbra, Portugal; 8 University of Coimbra, Coimbra Institute for Clinical and Biomedical Research (iCBR), Faculty of Medicine, Coimbra, Portugal; 9 Center for Innovative Biomedicine and Biotechnology (CIBB), University of Coimbra, Coimbra, Portugal; 10 Institute of Pharmacology and Experimental Therapeutics, Faculty of Medicine, University of Coimbra, Coimbra, Portugal; 11 Association for Innovation and Biomedical Research on Light and Image (AIBILI), Coimbra, Portugal

**Keywords:** keratoconus, progression, tear fluid biomarkers, cytokines, inflammation

## Abstract

**Purpose:**

Keratoconus is traditionally classified as a noninflammatory corneal ectasia, despite growing evidence suggesting an underlying inflammatory component. This study evaluates whether patients with stable keratoconus exhibit persistent inflammatory activity in tear fluid compared to healthy controls.

**Methods:**

Cross-sectional case-control study. Keratoconus progression was evaluated using tomographic and clinical criteria. Tear fluid samples were collected under standardized conditions and concentrations of nine cytokines (IFN-γ, IL-1β, IL-2, IL-4, IL-6, IL-10, IL-12p70, IL-17A, and TNF-α) were quantified using a multiplex assay. Group comparisons, correlation analyses, and receiver operating characteristic (ROC) curves were performed to evaluate cytokine expression and network behavior.

**Results:**

A total of 23 stable keratoconus patients and 25 age-matched healthy controls were included. The stable keratoconus group exhibited significantly elevated levels of tear fluid inflammatory cytokines compared to controls (all *P* < 0.05, except IL-2). Spearman correlation heatmaps revealed a coordinated cytokine network in the keratoconus group, suggesting persistent immunological activation despite clinical quiescence. No significant correlations were observed between cytokine levels and keratoconus staging indices. ROC analysis indicated moderate discriminatory performance of IL-6 (area under the curve = 0.68).

**Conclusions:**

Even clinically stable keratoconus is associated with a distinct proinflammatory tear fluid cytokine profile, challenging the traditional paradigm of keratoconus as a noninflammatory disease. These findings highlight the potential utility of tear fluid-based inflammatory biomarkers in keratoconus and suggest inflammation may persist independently of clinical progression.

**Translational Relevance:**

This study highlights the potential role of tear-based inflammatory biomarkers for monitoring disease activity, understanding keratoconus pathophysiology and guiding adjunctive anti-inflammatory therapies in keratoconus beyond structural stabilization.

## Introduction

Keratoconus is a progressive corneal disease characterized by stromal thinning and cone-shaped protrusion due to reduced biomechanical integrity, leading to irreversible visual impairment. Historically it has been described as a noninflammatory corneal thinning, although this description remains controversial.^1^ Despite the absence of clinical signs of inflammation, emerging evidence increasingly highlights the inflammatory component in its pathophysiology, with increased proteinase activity and decreased expression of proteinase inhibitors resulting in loss of biochemical stability.^2^^,^^3^ This is supported by multiple studies showing altered levels of cytokines, chemokines, and immune mediators both in the tear fluid and corneal epithelial cells of keratoconus patients compared to healthy controls.^4^^–^^6^ Apart from the corneal microenvironment, this was also evident in systemic biomarkers of inflammation (hair cortisol, neutrophil-lymphocyte ratio and platelet-lymphocyte ratio) and in variants in the interleukin (IL)-1 gene cluster.^7^^–^^9^ Atopy and allergic conjunctivitis are common comorbidities in patients with keratoconus and are strongly associated with eye rubbing, a known mechanical and inflammatory trigger in disease pathogenesis.^10^ Eye-rubbing is also associated with increased levels of IL-6 and tumor necrosis factor α (TNF-α).^11^

Despite these insights, a critical gap persists in understanding the link between inflammation and disease progression, and its role in keratoconus cases that are clinically stable. Existing studies either do not include an assessment of the status of the disease, or focus on progressive keratoconus and after corneal cross-linking, with limited attention to patients whose corneal tomography has remained unchanged.^4^^–^^6^^,^^12^^,^^13^ This gap raises an important question: Does a stable tomographic profile coincide with a normalized inflammatory environment, or could a dysregulated inflammatory environment persist despite clinical quiescence?

This innovative study, specifically targeting the immunological profile of non-progressive keratoconus, addresses this question by evaluating tear inflammatory cytokine levels in keratoconus patients with stable disease, compared to healthy controls, using multiplex cytokine profiling. By focusing on a stable clinical phenotype, this study aims to elucidate whether persistent low-grade inflammation may be an underlying factor in keratoconus pathophysiology, even in the absence of active progression, for which a consensual definition and better understanding is still a critical unmet need.^1^ This study addresses an important knowledge gap: whether keratoconus patients with clinically and tomographically stable disease exhibit persistent immunological activation, despite the absence of structural progression. Clarifying this could inform our understanding of disease pathophysiology and support the potential role of inflammatory strategies in keratoconus management.

## Methods

### Patients

This cross-sectional study included participants recruited at the Coimbra Local Health Unit (Coimbra, Portugal). Institutional ethics approval was obtained from the Faculty of Medicine of the University of Coimbra (CE-045/2021) and the study adhered to the tenets of the Declaration of Helsinki. Keratoconus patients were diagnosed according to the recommendations by the Global Consensus on Keratoconus and Ectatic Diseases using Pentacam tomography (Oculus Optikgerate GmbH).^1^ Recruitment occurred from November 30, 2023, to December 31, 2024.

Keratoconus progression was classified according to predefined criteria previously published by our group, based on a clinical visit and same-day corneal tomography: documentation of 2 or more specific parameters, including increase in maximum keratometry or mean keratometry of more than 1 diopter (D); decrease in central corneal thickness exceeding 5% or 20 µm; increase in manifest myopia, astigmatism, or spherical equivalent superior to 1 D; increase in posterior elevation exceeding 15 µm; and decrease of more than 1 Snellen line of corrected distance visual acuity (CDVA). The protocol for intervals between visits was age-dependent: every three months for patients under 18 years, every six months for those between 18 and 26 years, and annually thereafter.^14^ Patients were deemed progressive if the corneal changes were above the threshold progression criteria in two consecutive visits. Otherwise, they were considered stable and consecutively invited to participate in the “stable keratoconus group.” As detailed in the informed consent submitted to ethics approval, patients were informed that their decision to participate in the study, whether affirmative or not, would have no impact on their clinical follow-up or the proposed treatment. If both eyes of the same patient fulfilled the inclusion criteria, the right eye was chosen. Only one eye per patient was included.

Patients with previous ocular surgeries such as corneal crosslinking or other corneal or ocular comorbidities were excluded, including pellucid marginal degeneration or post-laser vision correction ectasia. These exclusion criteria applied both to the study eye and the fellow eye, meaning that if one eye fulfilled the inclusion criteria but the fellow eye had a previous corneal crosslinking or corneal transplant, this case would be excluded.

A control group of healthy participants were invited. History of eye rubbing and previous ocular diseases or surgeries were exclusion criteria. Inclusion criteria for both groups was an age range between 18 and 35 years of age.

For both groups, other known systemic diseases or use of topical or systemic medication in the 180 days before the inclusion (including steroids, nonsteroidal anti-inflammatory or anti-allergic drugs), pregnancy, systemic/ocular allergy or infection were exclusion criteria for both groups. Only artificial tears were allowed. Patients using contact lenses were instructed to discontinue their use for four weeks. Patients with active allergic eye disease or dry eye were excluded, including signs of eyelid eczema, redness or swelling, tarsal papillary hypertrophy or limbal Horner-Trantas, conjunctival staining, corneal punctate epithelial keratitis, epithelial defects or neovascularization, ocular pain, itching, red eye, or foreign body sensation.

All patients performed same-day Pentacam corneal tomography. A comprehensive clinical chart was built, including ID number, date of birth, laterality, manifest refraction, and a list of Pentacam-derived corneal metrics.

**Figure 1.**
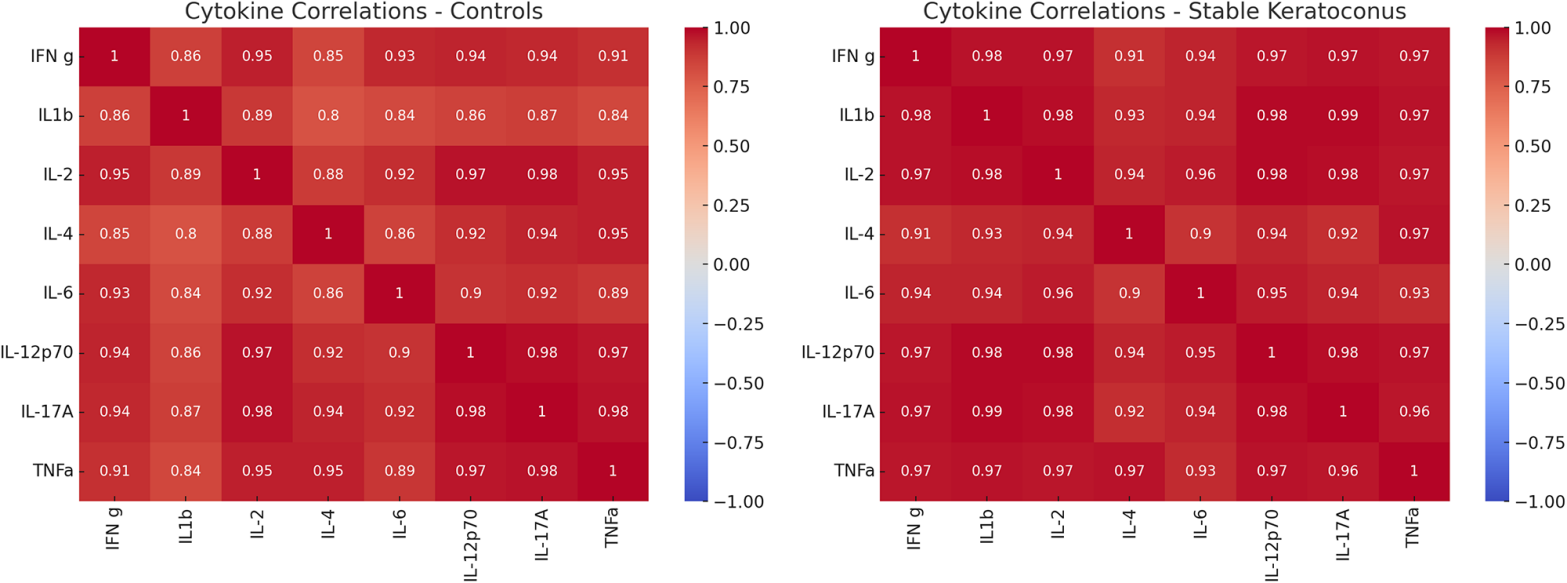
Spearman correlation heatmaps of tear fluid cytokine concentrations in healthy controls (*left*) and stable keratoconus patients (*right*). The color scale represents the strength and direction of correlation (ρ), from −1 (perfect negative) to +1 (perfect positive).

**Figure 2.**
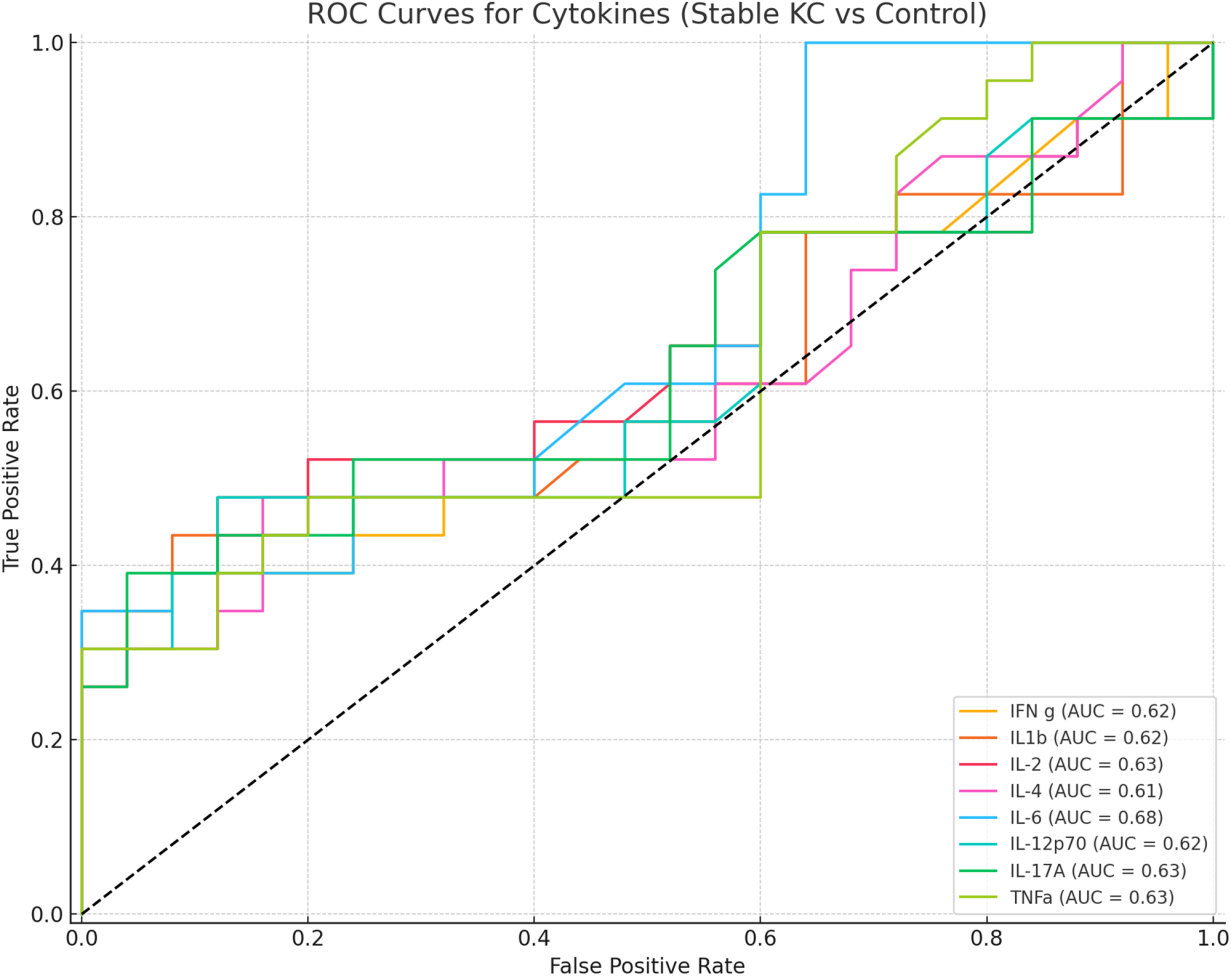
ROC curves illustrating the discriminatory performance of individual tear fluid cytokines in distinguishing stable keratoconus patients from healthy controls. Each curve represents a single cytokine, with the AUC reported in the legend.

### Tear Fluid Collection and Protein Elution

Tear fluid samples were collected between 10:00 AM and 1:00 PM to minimize diurnal variability, before the application of any topical medication (such as oxybuprocaine hydrochloride or iodopovidone) or any ocular procedure such as fluorescein vital staining or Schirmer test. Collection was performed using Schirmer filter paper strips (Dina strip Schirmer-Plus; Dina-Hitex, Bucovice, Czech Republic). Sterile 35 mm Schirmer strips were placed at the junction of the lateral and middle thirds of the lower eyelid and kept in place for five minutes, during which time participants were instructed to close their eyes. The wetted portion of each strip was trimmed and immediately placed into 2 mL Eppendorf tubes, which were stored at −80°C until processing. Protein extraction from the tear fluid samples was conducted with modifications to previously described protocols.^15^^,^^16^ Briefly, frozen strips were thawed, cut in small pieces, and incubated in 150 µL of 0.9% NaCl with 1× protease inhibitor cocktail (Roche, Basel, Switzerland) for three hours on ice to elute tear proteins. The resulting eluates were aliquoted and stored at −80°C until further analysis. Total protein concentration was determined using the Pierce BCA Protein Assay kit (Thermo Fisher Scientific, Rockford, IL, USA), with bovine serum albumin as the standard.

### Multiplex Cytokine Analysis in Tear Fluid

A panel of nine cytokines (IFN-γ, IL-1β, IL-2, IL-4, IL-6, IL-10, IL-12p70, IL-17A [CTLA-8], and TNF-α) was quantified using the ProcartaPlex Human High Sensitivity 9-Plex ProcartaPlex Panel (Thermo Fisher Scientific, Vienna, Austria), based on Luminex xMAP technology. Tear fluid samples were thawed on ice, vortexed briefly, and spun in a centrifuge at 10,000*g* for 10 minutes to remove particulate matter. The supernatants were transferred to new microcentrifuge tubes for analysis. A total of 25 µg of protein from each sample was used, following the manufacturer's protocol. Briefly, magnetic beads conjugated with capture antibodies were incubated overnight at 4°C with either standards or samples. The wells were then incubated sequentially with a biotinylated detection antibody mixture (30 minutes) and streptavidin-phycoerythrin conjugate (30 minutes), with thorough washing steps between incubations. After the addition of the reading buffer, samples were analyzed with the Luminex instrument. Standard curves were generated by using the reference cytokine samples supplied by the manufacturer. Raw data were analyzed by ProcartaPlex Analyst 1.0 Software to obtain analyte concentrations in tear fluid samples.

### Sample Size Calculation

Sample size calculations were made targeting IL-6. The choice of IL-6 among the different immune mediators was based on its frequent presence in the literature and for its pivotal role in chronic immune responses, particularly in keratoconus and two of its major risk factors: eye rubbing and atopy.^2^^,^^3^ Among the different published studies, the data used for sample size calculation was collected from the most recent study (2024), published in one of the most reputed scientific journals (*Ophthalmology*, by the American Academy of Ophthalmology) and with a large sample size of 100 eyes.^5^

IBM SPSS Statistics (v29.0.2.0 for Mac; IBM Corp.) was used assuming a significance level (α) of 0.05 and a desired power (1 − β) of 0.95, inputting the IL-6 concentrations mean (pg/mL) ± standard deviation for both the keratoconus patients (5.78 ± 2.88) and healthy controls (1.37 ± 1.01) in a two-sided analysis.^5^ The resulting sample size required to detect a statistically significant difference between the two groups was eight participants per group. Because the study did not include an assessment of the disease status (stable/progressive) we doubled the sample size estimates to account for uncertainty.

### Statistical Analysis

For statistical analysis, GraphPad (GraphPad, San Diego, CA, USA) and IBM SPSS Statistics (v29.0.2.0 for Mac; IBM, Armonk, NY, USA) were used. Normality was checked by the Kolmogorov-Smirnov test. When the sample followed a normal distribution, a parametric test was preferred and a *P* value ≤ 0.05 was considered statistically significant.

Each analyte's lower limit of quantification (LLOQ) was defined based on the corresponding Certificate of Analysis (Lot no. 332152-007). The LLOQs (in pg/mL) were as follows: IFN-γ (1.26), IL-1β (0.28), IL-10 (0.17), IL-12p70 (0.77), IL-17A (0.30), IL-2 (0.88), IL-4 (1.29), IL-6 (1.29), and TNF-α (0.62). Values falling below the LLOQ were considered left-censored. For statistical analyses, these values were inputted using LLOQ/2, a common conservative approach for handling left-censored biomarker data.^17^ Cytokines with more than 20% of values below the LLOQ were analyzed as binary variables (detectable vs. undetectable). Group comparisons were performed using the χ^2^ test (or Fisher's exact test where appropriate).

To evaluate the internal structure and co-regulation patterns of tear cytokines, Spearman correlation coefficients were computed separately for the stable keratoconus and control groups. Correlation matrices were visualized as heatmaps using the Seaborn Python library (v0.11.2), with values ranging from −1 to 1 and color-coded for interpretability. To quantitatively compare the correlation structures between groups, two summary metrics were calculated. Mean absolute correlation (MAC) is the average of the absolute values of all pairwise Spearman correlation coefficients (excluding the diagonal), providing a single value representing overall correlation strength within each group. Network Density is defined as the proportion of cytokine pairs with |ρ| ≥ 0.5 (absolute value of the Spearman correlation coefficient).

To evaluate the ability of individual cytokines to discriminate between stable keratoconus patients and healthy controls, receiver operating characteristic (ROC) curve analysis was performed. The ROC curve plots sensitivity (true positive rate) against 1-specificity (false-positive rate) across all possible concentration thresholds for classifying a subject as keratoconus or control. The area under the curve (AUC) was calculated for each cytokine as a summary measure of its discriminatory performance. AUC values range from 0.5 (no discrimination) to 1.0 (perfect discrimination). Graphic display of a single ROC plot with multiple cytokines was performed with Python's scikit-learn and matplotlib libraries.

## Results

A total of 48 patients were recruited in this study, 23 stable keratoconus patients and 25 healthy controls. Overall mean age was 26.5 ± 4.9 years, with no differences between groups (stable keratoconus: 26.4 ± 6.2; controls: 26.6 ± 3.4; *P* = 0.890). Table 1 includes a descriptive statistical characterization of both study groups regarding a wide variety of Pentacam corneal metrics meaningful in keratoconus, including pachymetry, anterior and posterior corneal surfaces, as well as composite indexes useful for keratoconus staging (ABCD) and screening (BAD-D). As expected, there were significant differences between groups.

**Table 1.** Demographic and Tomographic Characterization of Both Study Groups

	Controls (*n* = 25)	Stable Keratoconus (*n* = 23)	*P* Value
Age, years	26.6 ± 3.4	26.4 ± 6.2	0.890
Mean keratometry (D)	43.2 ± 1.7	47.0 ± 2.9	<0.001
Thinnest pachymetry (µm)	545.7 ± 32.5	473.5 ± 44.0	<0.001
Maximum keratometry (D)	44.0 ± 1.7	53.9 ± 5.7	<0.001
ISV	14.9 ± 5.3	72.7 ± 35.2	<0.001
IVA	0.1 ± 0.1	0.8 ± 0.4	<0.001
KI	1.0 ± 0.02	1.2 ± 0.1	<0.001
CKI	1.0 ± 0.01	1.1 ± 0.1	<0.001
IHA	3.4 ± 2.3	30.3 ± 21.8	<0.001
Rmin	7.7 ± 0.3	6.3 ± 0.6	<0.001
TKC	—	2.0 ± 0.8	—
KISA	8.4 ± 9.7	1524.0 ± 2573.3	0.010
IS	0.03 ± 0.4	4.9 ± 3.7	<0.001
A	0.1 ± 0.3	2.2 ± 1.7	<0.001
B	0.3 ± 0.6	3.5 ± 2.3	<0.001
C	0.4 ± 0.5	1.8 ± 1.2	<0.001
BAD-D	1.2 ± 0.7	7.1 ± 3.8	<0.001

BAD-D, Belin/Ambrósio Enhanced Ectasia Display; CKI, central keratoconus index; D, diopters; IHA, index of height asymmetry; IS, inferior-superior dioptric asymmetry; ISV, index of surface variance; IVA, index of vertical asymmetry; KI, keratoconus index; Rmin, radius minimum; TKC, Topographic Keratoconus Classification.

Table 2 reports the comparisons of cytokine concentration in tear fluid samples for both stable keratoconus and healthy control groups. Significant differences were observed between groups for all cytokines, except for IL-2, which did not reach statistical significance.

**Table 2.** Cytokine Concentration (pg/mL) in Tear Fluid Samples for Both Stable Keratoconus and Healthy Control Groups

	Cytokine Concentration (pg/mL)		
	Controls (*n* = 25)	Stable Keratoconus (*n* = 23)	*P* Value
IFN-γ	4.34 ± 3.48	8.31 ± 8.01	0.036
IL-1β	6.01 ± 3.14	9.49 ± 7.15	0.040
IL-12p70	8.36 ± 6.07	15.21 ± 13.45	0.033
IL-17A	2.71 ± 1.95	5.04 ± 4.21	0.022
IL-2	6.15 ± 2.96	8.45 ± 4.88	0.059
IL-4	3.64 ± 2.24	6.15 ± 5.28	0.044
IL-6	7.71 ± 3.78	12.00 ± 7.38	0.018
TNF-α	3.77 ± 3.08	7.94 ± 8.65	0.038

Values are presented as mean ± standard deviation. *P* values refer to comparisons between groups using either the independent-samples *t*-test or the Wilcoxon rank-sum test, according to the results of normality testing.

Values below the LLOQ were observed rarely: 12.5% (n = 6, three in each group) for IFN-γ, 4.17% (n = 2, both in the keratoconus group) for IL-17A, 2.08% (n = 1 in the control group) for TNF-α and never for the remaining cytokines. The exception was IL-10, for which 56.25% (n = 27) of values were below the LLOQ. Therefore IL-10 was treated as a binary variable. The difference in detection rates between groups was not significant (χ^2^ = 1.273, *P* = 0.259).

Only considering the keratoconus group, no significant correlations were found between the concentrations of the evaluated cytokines and continuous variables validated as markers of disease staging (parameters A, B, and C of the ABCD criteria). Similarly, no significant association was observed with maximum keratometry.

To explore broader inflammatory patterns beyond individual cytokine comparisons, Spearman correlation heatmaps were generated for each group to evaluate cytokine co-regulation (excluding IL-10 because of high undetectable rates) (Fig. 1). Stable keratoconus patients displayed stronger positive correlations among multiple cytokines, particularly IL-6, IL-1β, and TNF-α, indicating a more coordinated inflammatory network. Spearman correlation matrices were compared using MAC as a summary measure of overall cytokine co-regulation. The stable keratoconus group demonstrated higher MAC (0.957) compared to controls (0.910), indicating stronger and more coordinated cytokine expression. Both groups exhibited complete network density (100% of cytokine pairs with |ρ| ≥ 0.5).

To assess the discriminatory capacity of individual cytokines between stable keratoconus and control groups, ROC curves were generated for each analyte (Fig. 2). Among the eight cytokines analyzed, IL-6 demonstrated the highest area under the curve (AUC = 0.68), followed by IL-17A (AUC = 0.63), IL-2 (AUC = 0.63), TNF-α (AUC = 0.63), IL-1β (AUC = 0.62), IL-12p70 (AUC = 0.62), IFN-γ (AUC = 0.62), and IL-4 (AUC = 0.61).

## Discussion

The tear fluid inflammatory profile of patients with stable keratoconus, defined by stringent tomographic and clinical criteria, was evaluated in this study. Both groups were similar in terms of age, known both to generally influence the immune response and specifically the effect of some immune mediators on the severity of keratoconus.^18^ Time of day of the tear fluid collection was also kept consistent, because circadian rhythms can influence both the immune system activity and the tear fluid composition.^19^^–^^21^ Results demonstrate significantly elevated concentrations across all pro- and anti-inflammatory cytokines in the tear fluid of stable keratoconus patients, suggesting the presence of a sustained, low-grade inflammatory state even in the absence of clinical progression. Only IL-2 did not reach the conventional threshold for statistical significance (*P* = 0.059), although it trended in the same direction. The selected cytokine panel was based on prior literature identifying these markers as relevant to keratoconus or ocular surface inflammation, as well as their inclusion in a validated multiplex assay compatible with tear fluid samples.^4^^–^^6^^,^^22^ This panel includes cytokines spanning both pro-inflammatory (e.g., IL-1β, IL-6, TNF-α, IL-17A) and regulatory/anti-inflammatory (e.g., IL-4, IL-10) pathways.

There is vast research challenging the notion of keratoconus as a “noninflammatory” disease. Numerous studies have identified altered cytokine expression in the tear fluid and corneal epithelium of keratoconus patients—particularly IL-6, TNF-α, and IL-1 family members—supporting a model of localized inflammation contributing to stromal degradation and extracellular matrix remodeling.^2^^,^^3^ Enzymatic imbalance with dysregulated proteolysis, particularly mediated by matrix metalloproteinases (which have a complex bidirectional interplay with interleukins), also has a role in corneal stromal degradation.^23^ Markers of systemic inflammation, measured by hair cortisol or neutrophil-to-lymphocyte ratio, are also elevated in keratoconus, which confirm that inflammation is not limited to the corneal microenvironment.^5^^,^^7^^,^^9^^,^^24^ Whether the altered cytokine composition of tear fluid observed in keratoconus is a downstream consequence of epithelial alterations or contributes directly to the disease pathogenesis has yet to be determined. Additionally, despite the elevated levels of inflammatory markers, clinical and histological evidence provides limited support for the presence of inflammation, as there is no significant cellular infiltration or neovascularization.^3^ As a cofounding factor, eye rubbing, a major environmental risk factor for keratoconus, is associated with increased levels of tear fluid pro-inflammatory mediators such as IL-6, TNF-α, and MMP-9.^25^ However, eye rubbing does not invariably result in keratoconus development, suggesting that inflammation alone is insufficient to drive the disease process. This observation supports the idea of a complex interplay between genetic susceptibility, environmental triggers, and biochemical dysregulation in keratoconus pathogenesis.

The relationship between corneal microenvironment and keratoconus progression remains poorly understood, in part because progression is currently solely based on tomographic/structural metrics that might not fully capture underlying biological activity, and also because available studies do not assess disease progression status, as detailed in a recent meta-analysis.^26^ An exception to the cross-sectional literature is the study by Fodor et al.,^27^ who conducted a one-year longitudinal analysis, in which nerve growth factor and IL-13 were able to successfully predict the progression of keratoconus. Results from the present study suggest that the inflammatory imbalance in clinically non-progressive cases persist beyond the active/progressive phase of disease, reinforcing the idea that inflammation might not merely be a secondary response to biomechanical changes but could represent a constitutive feature of the disease process, regardless of its clinical status. This altered immunological state does not necessarily normalize with clinical stabilization, implying that progression and inflammation may follow partially independent trajectories. This idea might be supported by the lack of correlation between cytokines’ concentration and continuous variables validated for disease staging. Santhiago et al.^5^ also did not find an association between the increased levels of inflammatory markers and the severity of keratoconus.

A brief note on IL-10 is needed. Although there was a significant number of samples below the threshold criteria for quantification, results fall in agreement with the existing literature. A recent meta-analysis also reported no differences in tear fluid IL-10 concentration between keratoconus and healthy controls.^26^ Given IL-10’s primarily role as an anti-inflammatory cytokine, it testifies the complex imbalance of proinflammatory and anti-inflammatory factors in keratoconus. Interestingly, IL-4, also primarily regarded as an anti-inflammatory cytokine, is essential for the differentiation of naïve CD4+ T cells into Th2 cells, which are central to the allergic inflammatory response.^28^ This pro-allergic pathway is particularly relevant considering that atopy and ocular allergy are known risk factors for keratoconus.^1^ The presence of elevated IL-4 levels in stable keratoconus patients align with this immunological context.

The use of Spearman correlation matrices further revealed stronger and more coordinated cytokine co-regulation in the stable keratoconus group compared to healthy controls, particularly involving IL-6, IL-1β, and TNF-α. Rather than a disrupted cytokine environment, these findings reflect an active, coordinated low-grade inflammatory response that persists despite the absence of tomographic progression. Additionally, ROC curve analysis was conducted to explore the diagnostic utility of individual cytokines. While none of the cytokines exhibited strong classification performance on their own, the results suggest that selected inflammatory mediators, particularly IL-6, may contribute to a distinct immunological signature in stable keratoconus when compared to healthy controls, highlighting its potential as biomarker for disease presence.

These findings have potential therapeutic implications. The current management of keratoconus predominantly targets biomechanical stabilization, particularly via corneal crosslinking. The persistence of inflammatory mediators in stable keratoconus raises the question of whether anti-inflammatory treatments might benefit this subgroup. Cyclosporine A, for instance, has been shown to suppress MMP-9 and key inflammatory cytokines in tear fluid and could potentially mitigate subclinical inflammation in keratoconus.^22^ Further studies are needed to assess whether modulating this persistent inflammatory milieu can influence subclinical progression or even delay the onset of tomographic changes in high-risk patients.

This study has several strengths, replicating similar studies but including an innovative assessment of stable keratoconus with strict phenotypic characterization, standardized tear fluid collection, processing and analysis protocols, and the use of a highly sensitive multiplex cytokine assay. However, certain limitations must be acknowledged. The cross-sectional design precludes temporal assessment of cytokine trends and causal inferences. Although the sample size was statistically powered for IL-6, this study may be underpowered to detect smaller differences in other cytokines. To account for potential variability in other cytokines and to enhance the robustness of the findings, the sample size was more than doubled compared to the minimum requirements after sample size calculations. Nonetheless, larger cohorts would further strengthen the generalizability and statistical power of results across all cytokines, and broader panels including chemokines and growth factors might be useful. Longitudinal studies correlating cytokine shifts with eventual topographic progression are warranted to further delineate whether these markers are predictive, epiphenomenal, or therapeutic targets. Although the initial study plan included a third group of progressive keratoconus, these patients were recruited and tears collected in a different clinical context, including measurements before and six months after corneal crosslinking, as part of a separate line of research in our group.^29^ Given the heterogeneity in disease status and timing of samples collection and analysis, to preserve methodological consistency, this article focuses on healthy controls and clinically stable keratoconus patients. Future studies are planned to prospectively collect longitudinal data to clarify the inflammatory dynamics across different stages of disease activity.

Additionally, although patients with overt allergic eye disease or dry eye were excluded, similar to the exclusion criteria used by previous studies^5^, subclinical immune influences cannot be entirely ruled out and must be acknowledged as a limitation and source of potential confounding. Whereas eye rubbing is recognized as a contributor to ocular surface inflammation and is closely linked with keratoconus, its frequency is often underestimated because of the unreliability of self-reported data.^30^ Recent innovative artificial intelligence–based methods using conventional smartwatches have emerged for the quantitative assessment of eye rubbing and may be included in future studies.^31^ Also, intentionally the recruited clinically stable patients were in an age range when disease progression remained plausible, given the natural history of keratoconus, which typically progresses during the second to fourth decades of life. These findings raise the question of whether a similar proinflammatory milieu persists in older keratoconus patients beyond the typical progression age.

In conclusion, results from this study provide compelling evidence that even clinically stable keratoconus is associated with a persistently dysregulated tear fluid cytokine profile characterized by elevated levels of proinflammatory mediators. This supports the concept of ongoing immune activation in keratoconus, even in the absence of clinical progression, and reinforces the evolving paradigm that inflammation, far from being absent, may be a central component of keratoconus pathogenesis. A more nuanced understanding of the interplay between biomechanical weakness and immunological dysregulation may help redefine therapeutic goals in keratoconus beyond structural stabilization alone.

## References

[1] Gomes J, Tan D, Rapuano C, et al. Global consensus on keratoconus and ectatic diseases. *Cornea*. 2015; 34: 359–369. 25738235 10.1097/ICO.0000000000000408

[2] Wisse RPL, Kuiper JJW, Gans R, Imhof S, Radstake T, Van Der Lelij A. Cytokine expression in keratoconus and its corneal microenvironment: a systematic review. *Ocular Surface*. 2015; 13: 272–283. 26235733 10.1016/j.jtos.2015.04.006

[3] Galvis V, Sherwin T, Tello A, Merayo J, Barrera R, Acera A. Keratoconus: an inflammatory disorder? *Eye (Lond)*. 2015; 29: 843–859. 25931166 10.1038/eye.2015.63 PMC4506344

[4] Santos A, Filho J, Cenedeze M, et al. Increased inflammatory mediators in the ocular surface tissue in keratoconus. *Mol Vis*. 2024; 30: 279–288. 39563679 PMC11575844

[5] Santhiago MR, Stival LR, Araujo DC, et al. Relationship of inflammatory mediators (interleukin and cortisol concentrations) with corneal epithelial quantifiable metrics. *Ophthalmol Sci*. 2024; 5(1): 100624 39624797 10.1016/j.xops.2024.100624 PMC11609233

[6] Shetty R, Deshmukh R, Ghosh A, Sethu S, Jayadev C. Altered tear inflammatory profile in Indian keratoconus patients—The 2015 Col Rangachari Award paper. *Indian J Ophthalmol*. 2017; 65: 1105–1108. 29133633 10.4103/ijo.IJO_233_17 PMC5700575

[7] Toprak M, Altıntaş Ö, Sezer ÖY, Tuğan BY. Evaluation of neutrophillymphocyte ratio, plateletlymphocyte ratio, and mean platelet volume levels in pediatric keratoconus patients. *Saudi J Ophthalmol*. 2024; 38: 257–260. 39465031 10.4103/sjopt.sjopt_170_24 PMC11503983

[8] Doroodgar F, Alizadeh F, Niazi S, et al. Inflammatory and genomic interactions within keratoconus susceptible patients: a nationwide registered case–control study. *Eye Vis (Lond)*. 2024; 11: 40. 39354641 10.1186/s40662-024-00407-z PMC11446043

[9] Karaca EE, Özmen MC, Ekici F, Yüksel E, Türkoglu Z. Neutrophil-to-lymphocyte ratio may predict progression in patients with keratoconus. *Cornea*. 2014; 33: 1168–1173. 25255134 10.1097/ICO.0000000000000260

[10] Seth I, Bulloch G, Vine M, et al. The association between keratoconus and allergic eye diseases: a systematic review and meta-analysis. *Clin Exp Ophthalmol*. 2023; 51(4): O1–O16. 36882200 10.1111/ceo.14215

[11] Balasubramanian SA, Pye DC, Willcox MDP. Effects of eye rubbing on the levels of protease, protease activity and cytokines in tears: Relevance in keratoconus. *Clin Exp Optom*. 2013; 96: 214–218. 23496656 10.1111/cxo.12038

[12] Balmus IM, Alexa AI, Ciuntu RE, et al. Oxidative stress markers dynamics in keratoconus patients ’ tears before and after corneal collagen crosslinking procedure. *Exp Eye Res*. 2020; 190: 107897. 31836491 10.1016/j.exer.2019.107897

[13] Krok M, Wr E, Łach-wojnarowicz O, Bronikowska J, Dobrowolski D. Analysis of cytokine and chemokine level in tear film in keratoconus patients before and after corneal cross-linking (CXL) treatment. *Int J Mol Sci*. 2024; 25: 1052. 38256126 10.3390/ijms25021052 PMC10816198

[14] Gil P, Gil JQ, Dias M, et al. Comparative analysis of combined topography-guided photorefractive keratectomy and corneal crosslinking in progressive versus stable keratoconus. *Cornea*. 2024; 44: 983–991. 39083227 10.1097/ICO.0000000000003653

[15] Amorim M, Martins B, Caramelo F, et al. Putative biomarkers in tears for diabetic retinopathy diagnosis. *Front Med (Lausanne)*. 2022; 9: 873483. 35692536 10.3389/fmed.2022.873483 PMC9174990

[16] Cruz A, Queirós R, Abreu CM, et al. Electrochemical immunosensor for TNFα-mediated inflammatory disease screening. *ACS Chem Neurosci*. 2019; 10: 2676–2682. 30985099 10.1021/acschemneuro.9b00036

[17] Pleil JD . Imputing defensible values for left-censored “below level of quantitation” (LoQ) biomarker measurements. *J Breath Res*. 2016; 10(4): 045001. 27753432 10.1088/1752-7155/10/4/045001

[18] Kolozsvári BL, Petrovski G, Gogolák P, et al. Association between mediators in the tear fluid and the severity of keratoconus. *Ophthalmic Res*. 2013; 51: 46–51. 24247644 10.1159/000351626

[19] Benito MJ, González-García MJ, Tesón M, et al. Intra- and inter-day variation of cytokines and chemokines in tears of healthy subjects. *Exp Eye Res*. 2014; 120: 43–49. 24412438 10.1016/j.exer.2013.12.017

[20] Arroyo CA, Byambajav M, Fernández I, et al. Diurnal variation on tear stability and correlation with tear cytokine concentration. *Cont Lens Anterior Eye*. 2022; 45(6): 101705. 35562228 10.1016/j.clae.2022.101705

[21] Uchino E, Sonoda S, Kinukawa N, Sakamoto T. Alteration pattern of tear cytokines during the course of a day: diurnal rhythm analyzed by multicytokine assay. *Cytokine*. 2006; 33: 36–40. 16406556 10.1016/j.cyto.2005.11.013

[22] Shetty R, Ghosh A, Lim RR, et al. Elevated expression of matrix metalloproteinase-9 and inflammatory cytokines in keratoconus patients is inhibited by cyclosporine A. *Invest Ophthalmol Vis Sci*. 2015; 56: 738–750. 25648341 10.1167/iovs.14-14831

[23] di Martino E, Ali M, Inglehearn CF. Matrix metalloproteinases in keratoconus—too much of a good thing? *Exp Eye Res*. 2019; 182: 137–143. 30910610 10.1016/j.exer.2019.03.016

[24] Lenk J, Spoerl E, Stalder T, et al. Increased hair cortisol concentrations in patients with progressive keratoconus. *J Refract Surg*. 2017; 33: 383–388. 28586498 10.3928/1081597X-20170413-01

[25] Balasubramanian SA, Pye DC, Willcox MDP. Effects of eye rubbing on the levels of protease, protease activity and cytokines in tears: relevance in keratoconus. *Clin Exp Optom*. 2013; 96: 214–218. 23496656 10.1111/cxo.12038

[26] Zhang H, Cao X, Liu Y, Wang P, Li X. Tear levels of inflammatory cytokines in keratoconus: a meta-analysis of case-control and cross-sectional studies. *BioMed Res Int*. 2021; 2021(1): 6628923. 34631885 10.1155/2021/6628923 PMC8497143

[27] Fodor M, Vitályos G, Losonczy G, et al. Tear mediators NGF along with IL-13 predict keratoconus progression. *Ocul Immunol Inflamm*. 2021; 29: 1090–1101. 32130054 10.1080/09273948.2020.1716024

[28] Junttila IS . Tuning the cytokine responses: an update on interleukin (IL)-4 and IL-13 receptor complexes. *Front Immunol*. 2018; 9: 888 29930549 10.3389/fimmu.2018.00888 PMC6001902

[29] Teixeira B, Costa C, Sobral P, et al. Avaliação dos Mediadores Inflamatórios no Filme Lacrimal Antes e Depois do Cross-Linking Corneano. *Rev Soc Portuguesa Oftalmol*. 2024, 10.48560/rspo.33242.

[30] Nichani PAH, Solomon B, Trinh T, et al. Investigating the role of inflammation in keratoconus: a retrospective analysis of 551 eyes. *Eur J Ophthalmol*. 2022; 33: 35–43. 36154720 10.1177/11206721221125013 PMC9834323

[31] Drira I, Louja A, Sliman L, et al. Eye-rubbing detection tool using artificial intelligence on a Smartwatch in the management of keratoconus. *Transl Vis Sci Technol*. 2024; 13(12): 16. 10.1167/tvst.13.12.16 PMC11645754 39666356

